# Feeling the Burn: Combustion By-Products Put Kids at Risk

**DOI:** 10.1289/ehp.115-a504b

**Published:** 2007-10

**Authors:** Bob Weinhold

One of the most extensive studies of the link between lower respiratory diseases and ambient concentrations of polycyclic aromatic hydrocarbons (PAHs), a class of widespread air pollutants, has found that the chemicals are a potent problem for preschool-age children **[*EHP* 115:1510–1518; Hertz-Picciotto et al.]**. The team of researchers also found that fine particles (PM_2.5_) pose a respiratory risk to very young children.

PAHs and PM_2.5_ are generated primarily by combustion of petroleum products, coal, wood, tobacco, trash, fat, and other substances. The concentrations of PAHs and PM_2.5_ in the two Czech Republic districts studied, Prachatice and Teplice, were similar to those found in many U.S., European, and Asian cities.

The study involved 1,133 children up to age 4.5 years. The team examined pediatric charts to determine those whom a doctor had diagnosed with a lower respiratory disease of acute bronchitis, tracheitis, or laryngitis. These children were among 7,502 born from May 1994 to December 1998 who were evaluated in previous studies, providing substantial data on factors such as fuels used for home heating and cooking; birth weight; and maternal education, age at birth, and smoking history.

The current study collected information on breastfeeding, daycare attendance, and other factors that could either protect these children or increase their risk of respiratory disease. Pollution data were obtained from selected monitors in each district; readings were taken during each month anywhere from once each day to once every six days. This allowed analysis of all seasons and a wide range of intervals.

The team found a statistically significant link between bronchitis, the most commonly diagnosed illness, and elevated ambient PAHs, escalating 56% in children aged 2 to 4.5 years for each 30-day-average increase of 100 ng/m^3^. The increase was 29% for children under age 2 years. For each 30-day-average increase in PM_2.5_ of 25 μg/m^3^, bronchitis diagnoses rose 30% for children up to age 2 years. In children aged 2 to 4.5, it rose 23%. Some other pollutants that could play a role in respiratory illness, such as ozone, nitrogen oxides, sulfur dioxide, and metals, were not covered in the analysis. Certain weather variables, such as wind and humidity, also were not considered.

However, the study was strengthened by the use of a doctor’s diagnosis rather than parental reports or recalled incidents, and the likelihood of a sick child visiting a doctor was very high, given that health care was free and readily available. Consistency of diagnostic coding also appeared to be very good. The strengths of the study and the magnitude of the findings lead the authors to conclude that relatively short-term exposure to ubiquitous PAHs may pose a significant respiratory threat to children.

